# Nonlinear association between hematocrit and 3-month outcome after acute ischemic stroke: identification of threshold effects in males

**DOI:** 10.3389/fmed.2026.1721251

**Published:** 2026-02-04

**Authors:** Xiaomin Liang, Qian Wang, Zemao Xing, Shuiqing Gui

**Affiliations:** 1 Department of Critical Care Medicine, Shenzhen Second People’s Hospital, The First Affiliated Hospital of Shenzhen University, Shenzhen, Guangdong, China; 2 Department of Gastroenterology, The Second Affiliated Hospital, Shenzhen and Longgang District People’s Hospital of Shenzhen, School of Medicine, The Chinese University of Hong Kong, Shenzhen, Guangdong, China

**Keywords:** acute ischemic stroke, hematocrit, nonlinear relationship, secondary analysis, threshold effect

## Abstract

**Objective:**

Acute ischemic stroke (AIS) is a leading cause of disability and mortality worldwide. Hematocrit (HCT), the proportion of blood volume occupied by red blood cells, has been associated with outcome in AIS patients, but the relationship remains controversial. This study aimed to investigate the association between HCT levels and 3-month adverse outcome in South Korean patients with AIS, with a focus on potential sex differences.

**Methods:**

This secondary analysis utilized data from an existing South Korean prospective cohort study involving 1,896 AIS patients. HCT was assessed both as a continuous variable and categorized into quartiles. The outcome was 3-month adverse outcome, defined as a modified Rankin Scale score ≥ 3. Logistic regression models were employed to evaluate the relationship between HCT and adverse outcome in both the overall population and sex-stratified subgroups. Nonlinear associations were explored using generalized additive models and two-piecewise linear regression analysis.

**Results:**

Our study included 1,896 AIS patients, comprising 1,163 males (61.34%) and 733 females (38.66%). The age distribution was as follows: < 60 years (22.89%), 60–70 years (26.53%), 70–80 years (35.07%), and ≥ 80 years (15.51%). The probability of adverse outcome was 28.53%. Females were more likely to experience unfavorable outcome (35.20%) compared to males (24.33%). A nonlinear relationship was identified in the overall population, with HCT levels below 34.4% demonstrating a 15% risk reduction per 1% increase (OR = 0.85, 95% CI 0.78–0.92, *P* = 0.0001). In males, a similar nonlinear relationship was observed, with HCT levels below 36.4% (95% CI 32.2–38.6%) associated with a 19% risk reduction per 1% increase (OR = 0.81, 95% CI 0.74–0.88, *P* < 0.0001). However, no significant association was found in female patients.

**Conclusion:**

This study revealed a nonlinear, sex-specific relationship between HCT and 3-month outcome in AIS patients. In male patients, HCT levels below the threshold were inversely associated with adverse outcome, while no significant association was observed in female patients.

## 1 Introduction

Acute ischemic stroke (AIS) is a critical medical condition characterized by the sudden interruption of blood flow to a part of the brain, leading to neuronal injury and potential long-term disability or death (1). In 2021, AIS was the most prevalent category, accounting for 62.4–67.7% of worldwide stroke occurrences (1), with higher rates in China (86.8%) (2) and South Korea (76%) (3). The modified Rankin Scale (mRS) is frequently used as a prognostic predictor for stroke patients following an acute incident (4, 5). The mRS score ranges from 0 (no symptoms) to 6 (death), and adverse outcome is characterized by an mRS score ≥ 3, including varying degrees of disability (moderate to severe) and death (6, 7). Despite recent therapy improvements, between 1 month and 5 years following a stroke, around 40% of survivors were disabled (mRS score 3–5) (8). Thus, it is essential to identify risk factors for adverse outcome in AIS patients.

Hematocrit (HCT), the proportion of blood volume occupied by red blood cells, is a critical determinant of blood viscosity and, consequently, cerebral blood flow (5). HCT serves as a multifaceted factor in stroke prognosis, reflecting both vascular function and systemic inflammatory status (9, 10). The association between HCT levels and outcome in patients with AIS has garnered significant attention in recent years and has been reported inconsistently. Some studies have shown that increased HCT correlates with worse neurological outcome (11–13). Conversely, low HCT levels have also been implicated in poor outcome (5, 14, 15). The contradictory findings in previous literature primarily stem from three critical factors: temporal differences across treatment eras, significant patient population heterogeneity, and methodological limitations. Most prior investigations assumed linear associations between HCT levels and stroke outcome while neglecting potential nonlinear relationships. Evidence demonstrates substantial sex differences in functional recovery following AIS, emphasizing the necessity of sex-specific analyses (11, 16–19). The biological foundations for sex differences include hemorheological differences, hormonal influences, and distinct inflammatory response patterns (20–23).

Based on these observations, we hypothesized that the relationship between HCT and 3-month unfavorable outcome in AIS patients follows a nonlinear pattern with significant sex-specific effects. To test this hypothesis, we conducted a secondary analysis of an existing South Korean prospective cohort study to investigate both the nonlinear nature of the HCT-outcome relationship and its sex-specific differential effects in AIS patients.

## 2 Materials and methods

### 2.1 Study design

This study performed a secondary analysis of an existing South Korean prospective cohort study. The study employed data from a single-center prospective registry system in South Korea, which were obtained between January 2010 and December 2016 (24). The independent variable in this study was the HCT. The dependent variable was the 3-month outcome of AIS patients, classified as favorable or adverse.

### 2.2 Data source and study population

The raw data may be downloaded for free from Kang et al., titled “Geriatric nutritional risk index predicts poor outcomes in patients with acute ischemic stroke—Automated undernutrition screen tool” (24).^1^ The institutional review board of Seoul National University Hospital approved the study and waived patient consent (IRB NO. 1009-062-332). Our secondary analysis adhered to the Declaration of Helsinki and followed relevant regulations and guidelines.

**FIGURE 1:**
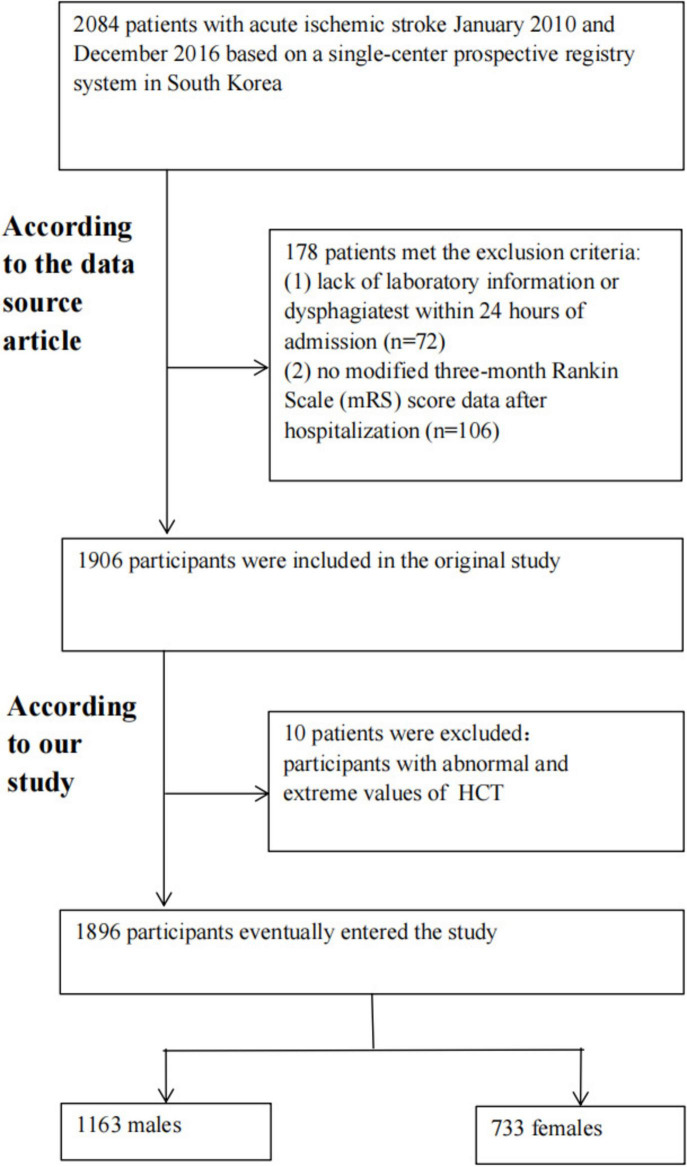
Participant selection procedure.

The original study included 2,084 South Korean patients with AIS who were hospitalized within 7 days of the commencement of their symptoms (24). Data from a single-center prospective registration system between January 2010 and December 2016 (24). Exclusion criteria were as follows: (1) No laboratory or dysphagia test findings within 24 h of admission; (2) No modified 3-month mRS score data after hospitalization (24). 1906 individuals were included in the initial data analysis. In our investigation, we further excluded people with abnormal and extreme HCT levels (below or larger than three standard deviations from the mean) (*n* = 10). Finally, our study has 1896 participants for secondary analysis. Figure 1 displays the participant selection procedure.

### 2.3 Variables

#### 2.3.1 HCT

The HCT was measured as a continuous variable and analyzed using both continuous and categorical approaches. For the categorical analysis, HCT was divided into quartiles based on the distribution in our study population: Q1: 23.60–36.90% (*n* = 474), Q2: 37.00–40.50% (*n* = 469), Q3: 40.60–43.80% (*n* = 466), Q4: 43.90–55.80% (*n* = 487).

#### 2.3.2 Three-month outcome in patients with AIS

The mRS score was used to assess 3-month outcome following AIS onset. Information was obtained through outpatient visits or structured telephone interviews. An adverse outcome was referred to as an mRS score ≥ 3, while a favorable outcome was determined as an mRS score ≤ 2 (24, 25).

### 2.4 Covariates

Covariates for our investigation were chosen based on available literature and clinical experience (1, 26–32). The following variables were used as covariates: (i) the continuous variables included red blood cell count (RBC), hemoglobin (HGB), white blood cell count (WBC), platelet count (PLT), total cholesterol (TC), triglyceride (TG), high-density lipoprotein-cholesterol (HDL-c), low-density lipoprotein-cholesterol (LDL-c), blood urea nitrogen (BUN), serum creatinine (Scr), fasting blood glucose (FBG), hemoglobin A1c (HBA1c), body mass index (BMI), international normalized ratio (PTINR), activated partial thromboplastin time (APTT), fibrinogen (FIB), and the National Institutes of Health Stroke Scale (NIHSS) score. (ii) The categorical variables were sex, age, stroke etiology, smoking status, previous stroke/transient ischemic attack (TIA), hypertension, diabetes, hyperlipidemia, atrial fibrillation, coronary heart disease (CHD), and mRS score at admission. Laboratory data (tests performed within 24 h of admission) were obtained from the electronic medical records (24). The NIHSS score at admission is used to determine the initial neurological severity (24). Classification of stroke subtypes depends on the trial of Org10172 in the treatment of acute stroke (TOAST) (24, 33).

### 2.5 Missing data processing

In the current study, missing data (N, %) were WBC (1, 0.05%), TG (9, 0.47%), and mRS at admission (1, 0.05%). Multiple imputations were used to manage missing covariant data (34). The imputation model included PLT, TC, HDL-c, LDL-c, BUN, Scr, FBG, HBA1c, PTINR, APTT, FIB, Age, Sex, BMI, Previous stroke/TIA, Hypertension, Diabetes, Hyperlipidemia, Smoking, Atrial fibrillation, CHD, NIHSS score at admission, Stroke etiology. Missing data analysis assumptions were based on the missing-at-random assumption (MAR) (35).

### 2.6 Statistical analysis

Individuals were divided into HCT quartile groups. For continuous variables, use the mean (standard deviation) or median (range) (non-normal distribution) presentation, and for categorized variables, use the percentage display. The differences between HCT groups were examined using one-way analysis of variance (normal distribution), the Kruskal-Wallis H test (skewed distribution), or the Chi-Squared technique (classified variables).

After screening for collinearity (HGB and RBC were excluded, Supplementary Table 1), the authors used multivariate logistic regression models to construct three distinct models to discover the link between HCT and 3-month adverse outcome in patients with AIS in the general population and sex subgroups. The models were as follows: (i) Crude model adjust for: None. (ii) Model I adjust for: Age and Sex. (iii) Model II adjust for: WBC, PLT, TC, TG, HDL-c, LDL-c, BUN, Scr, FBG, HBA1c, PTINR, APTT, FIB, Age, Sex, BMI, Previous stroke/TIA, Hypertension, Diabetes, Hyperlipidemia, Smoking, Atrial fibrillation, CHD, NIHSS score at admission, mRS at admission, Stroke etiology. Sex was not adjusted in sex subgroups. The data are shown as odds ratio (OR) and 95% confidence interval (CI).

Generalized additive models (GAM) and smooth curve fitting (penalized spline approach) were employed to investigate the nonlinear connection between HCT and 3-month unfavorable outcome. We also performed stratified analyses by sex to examine potential differences in the HCT-outcome relationship between males and females. If nonlinearity was discovered, we used a recursive approach to compute the inflection point before applying a two-piecewise linear regression model to both sides of the inflection point (36). The log-likelihood ratio test was performed to find the best model for capturing the relationship between HCT and the 3-month unfavorable outcome. The 95% CIs for the inflection points were calculated using bootstrap resampling (1,000 repetitions) (36).

A range of sensitivity analyses were performed to ensure the robustness of our findings. Patients with hypertension, Atrial fibrillation, and CHD have a much higher risk of unfavorable prognosis (29–32); hence, when investigating the link between HCT and 3-month poor outcome in sensitivity analyses, we excluded individuals with hypertension, Atrial fibrillation, and CHD. Moreover, by estimating E-value, we evaluated the possibility of undetected confounding between HCT and unfavorable outcome (37).

All findings were prepared using the STROBE statement (38). All statistical analyses were carried out with R software (version 4.2.0) and EmpowerStats (version 4.2). *P*-values < 0.05 (two-sided) were deemed statistically significant.

## 3 Results

### 3.1 Characteristics

**FIGURE 2:**
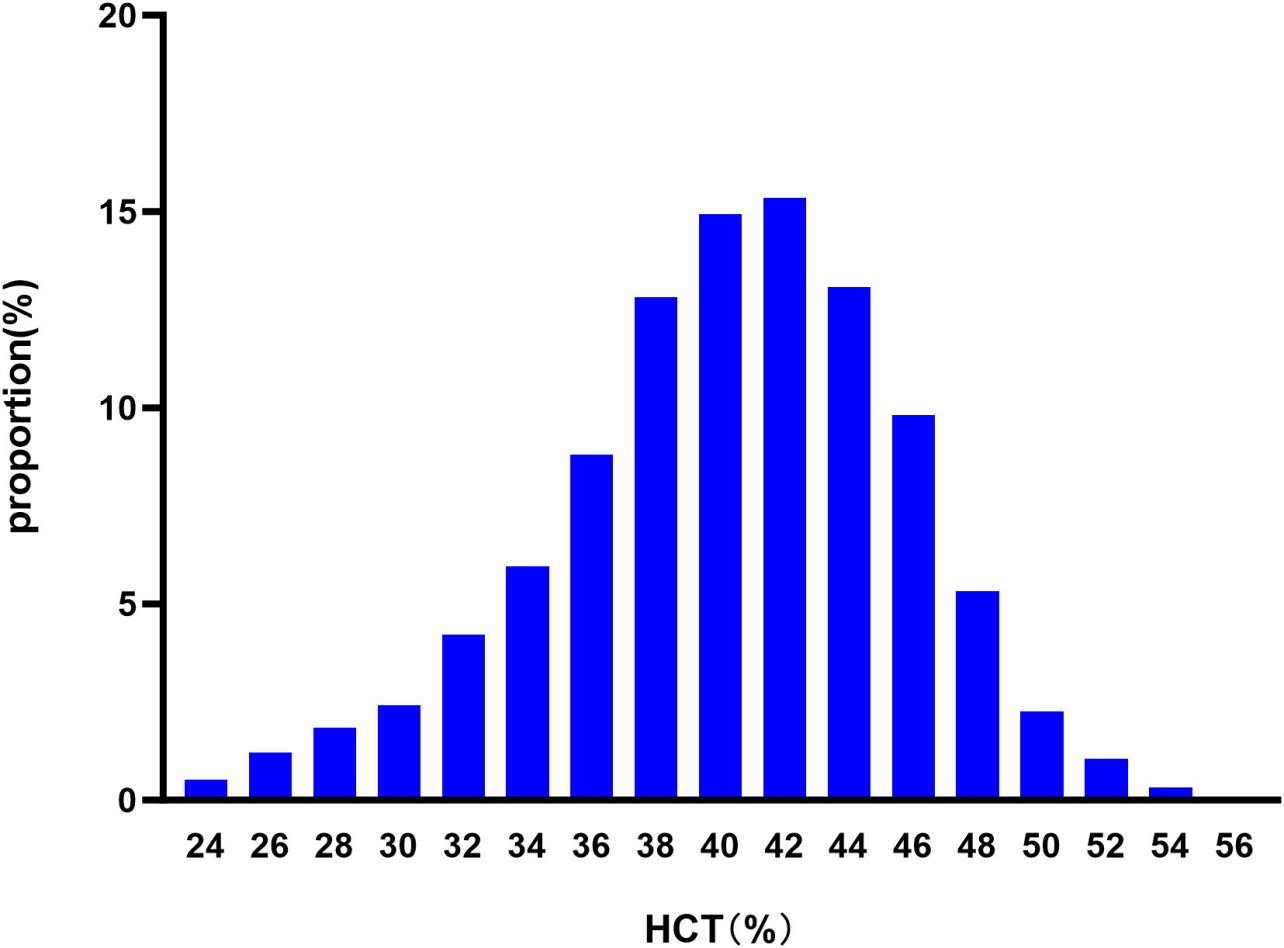
Distribution of HCT. The distribution ranged from 23.60 to 55.80%, with a mean of 40.18 ± 5.42.

Our study included 1,896 AIS patients, with 1,163 males (61.34%) and 733 females (38.66%). The age distribution was as follows: < 60 years (22.89%), 60–70 years (26.53%), 70–80 years (35.07%) and ≥ 80 years old (15.51%). The probability of adverse outcome was 28.53%. The distribution ranged of HCT from 23.60 to 55.80%, with a mean of 40.18 ± 5.42 (Figure 2). Patients were divided into four HCT quartiles. As HCT increased, the proportion of male and younger (<60 years) patients significantly increased. RBC, HGB, and HCT values also increased across quartiles. Lipid profiles showed higher TC, TG, and LDL-c, but lower HDL-c with rising HCT. Renal function indicators revealed decreasing BUN but increasing Scr with higher HCT. The proportions of prior stroke/TIA and diabetes decreased, while smoking increased across HCT quartiles. NIHSS score at admission decreased with higher HCT. The proportions of LAA and SVO etiologies increased, while CE decreased with rising HCT. The probability of adverse outcome decreased as HCT quartiles increased (Table 1).

**TABLE 1:** Participant characteristics by HCT quartile.

Variables	Overall	HCT (%) quartile				*P*
		Q1 (23.60–36.90)	Q2 (37.00–40.50)	Q3 (40.60–43.80)	Q4 (43.90–55.80)	
Participants	1,896	474	469	466	487	
Sex						< 0.001
Male	1,163 (61.34%)	202 (42.62%)	221 (47.12%)	318 (68.24%)	422 (86.65%)	
Female	733 (38.66%)	272 (57.38%)	248 (52.88%)	148 (31.76%)	65 (13.35%)	
Age (years)						< 0.001
<60	434 (22.89%)	70 (14.77%)	65 (13.86%)	122 (26.18%)	177 (36.34%)	
60 to < 70	503 (26.53%)	99 (20.89%)	123 (26.23%)	135 (28.97%)	146 (29.98%)	
70 to < 80	665 (35.07%)	202 (42.62%)	180 (38.38%)	157 (33.69%)	126 (25.87%)	
≥ 80	294 (15.51%)	103 (21.73%)	101 (21.54%)	52 (11.16%)	38 (7.80%)	
RBC (10^12/L)	4.33 ± 0.62	3.56 ± 0.43	4.21 ± 0.25	4.53 ± 0.24	5.02 ± 0.34	< 0.001
HGB (g/dL)	13.51 ± 1.94	10.95 ± 1.25	13.09 ± 0.57	14.26 ± 0.52	15.70 ± 0.86	< 0.001
HCT (%)	40.18 ± 5.42	32.96 ± 3.31	38.86 ± 1.01	42.17 ± 0.94	46.56 ± 2.21	< 0.001
WBC (10^9/L)	8.15 ± 2.88	7.88 ± 3.40	7.93 ± 2.58	8.18 ± 2.90	8.58 ± 2.54	< 0.001
PLT (10^9/L)	224.02 ± 71.00	222.50 ± 88.50	220.36 ± 63.39	222.98 ± 62.60	230.02 ± 66.04	0.169
TC (mg/dL)	179.53 ± 43.79	164.99 ± 43.44	176.88 ± 42.47	183.80 ± 41.90	192.17 ± 42.84	< 0.001
TG (mg/dL)	94.00 (70.00–130.00)	87.00 (63.00–114.75)	89.00 (67.00–125.50)	97.00 (73.00–136.00)	105.00 (79.00–142.00)	< 0.001
HDL-c (mg/dL)	44.24 ± 16.79	41.30 ± 19.13	45.71 ± 16.45	46.58 ± 15.93	43.47 ± 14.94	< 0.001
LDL-c (mg/dL)	104.34 ± 42.37	91.99 ± 42.92	103.59 ± 40.91	107.12 ± 41.44	114.42 ± 41.16	< 0.001
BUN (mg/dL)	16.00 (12.00–20.00)	17.50 (13.00–24.00)	16.00 (13.00–20.00)	15.00 (12.00–19.00)	15.00 (12.00–18.00)	< 0.001
Scr (mg/dL)	0.89 (0.74–1.08)	0.90 (0.71–1.25)	0.85 (0.70–1.07)	0.88 (0.74–1.02)	0.93 (0.80–1.08)	< 0.001
FBG (mmol/L)	99.06 ± 46.02	92.10 ± 48.40	99.93 ± 44.84	101.24 ± 44.60	102.91 ± 45.52	0.001
HBA1c (%)	5.80 (5.30–6.50)	5.90 (4.90–6.40)	5.90 (5.50–6.50)	5.80 (5.40–6.30)	5.80 (5.30–6.40)	0.071
PTINR	1.04 ± 0.33	1.07 ± 0.44	1.05 ± 0.30	1.03 ± 0.31	1.02 ± 0.25	0.052
APTT (sec)	30.81 ± 6.52	30.59 ± 8.31	30.75 ± 6.38	30.99 ± 5.57	30.92 ± 5.43	0.785
FIB (mg/L)	330.19 ± 91.73	346.23 ± 120.48	326.54 ± 80.78	322.53 ± 79.71	325.44 ± 77.30	< 0.001
BMI (kg/m^2)	23.51 ± 3.25	22.43 ± 3.16	23.10 ± 3.21	23.82 ± 3.10	24.66 ± 3.11	< 0.001
Previous stroke/TIA	401 (21.15%)	115 (24.26%)	114 (24.31%)	94 (20.17%)	78 (16.02%)	0.004
Hypertension	1,206 (63.61%)	316 (66.67%)	309 (65.88%)	283 (60.73%)	298 (61.19%)	0.118
Diabetes	608 (32.07%)	187 (39.45%)	178 (37.95%)	115 (24.68%)	128 (26.28%)	< 0.001
Hyperlipidemia	697 (36.76%)	150 (31.65%)	167 (35.61%)	176 (37.77%)	204 (41.89%)	0.010
Smoking	748 (39.45%)	114 (24.05%)	126 (26.87%)	218 (46.78%)	290 (59.55%)	< 0.001
Atrial fibrillation	406 (21.41%)	107 (22.57%)	111 (23.67%)	79 (16.95%)	109 (22.38%)	0.056
CHD	218 (11.50%)	60 (12.66%)	64 (13.65%)	52 (11.16%)	42 (8.62%)	0.080
NIHSS score at admission	3.00 (1.00–7.00)	4.00 (2.00–10.00)	4.00 (1.00–7.00)	3.00 (1.00–6.00)	3.00 (1.00–6.00)	< 0.001
mRS at admission						0.097
< 3	1,672 (88.23%)	412 (86.92%)	404 (86.14%)	423 (90.97%)	433 (88.91%)	
≥ 3	223 (11.77%)	62 (13.08%)	65 (13.86%)	42 (9.03%)	54 (11.09%)	
Stroke etiology						< 0.001
LAA	604 (31.86%)	135 (28.48%)	151 (32.20%)	157 (33.69%)	161 (33.06%)	
SVO	364 (19.20%)	69 (14.56%)	98 (20.90%)	98 (21.03%)	99 (20.33%)	
CE	492 (25.95%)	132 (27.85%)	130 (27.72%)	104 (22.32%)	126 (25.87%)	
Other determined	167 (8.81%)	80 (16.88%)	26 (5.54%)	30 (6.44%)	31 (6.37%)	
Undetermined	269 (14.19%)	58 (12.24%)	64 (13.65%)	77 (16.52%)	70 (14.37%)	
Probability of adverse outcome						< 0.001
No	1,355 (71.47%)	284 (59.92%)	330 (70.36%)	369 (79.18%)	372 (76.39%)	
Yes	541 (28.53%)	190 (40.08%)	139 (29.64%)	97 (20.82%)	115 (23.61%)	

Continuous variables: mean (standard deviation) or median (range). Categorized variables: N (%). HCT, hematocrit; AIS, Acute ischemic stroke; RBC, Red Blood Cell Count; HGB, Hemoglobin; WBC, White Blood Cell Count; PLT, Platelet Count; TC, Total Cholesterol; TG, Triglyceride; HDL-c, High-Density Lipoprotein-Cholesterol; LDL-c, Low-Density Lipoprotein-Cholesterol; BUN, Blood Urea Nitrogen; Scr, Serum Creatinine; FBG, Fasting Blood Glucose; HbA1c, Hemoglobin A1c; BMI, Body Mass Index; PTINR, International Normalized Ratio; APTT, Activated Partial Thromboplastin Time; FIB, Fibrinogen; TIA, Transient Ischemic Attack; NIHSS, National Institutes of Health Stroke Scale; CHD, Coronary Heart Disease; mRS, Modified Rankin Scale; LAA, large artery atherosclerosis; SVO, small vessel occlusion; CE, cardio embolism.

This study included 1,163 male and 733 female participants (Table 2). The results showed that compared to female participants, male participants had significantly higher levels of RBC, HGB, HCT, TG, BUN, Scr levels, BMI, and the smoking rate. In contrast, female participants had significantly higher PLT, TC, HDL-c, LDL-c levels, PTINR, and NIHSS score at admission compared to male participants. Additionally, the proportion of atrial fibrillation, hypertension, hyperlipidemia, and stroke etiology were significantly higher in female participants. Female patients were older than male patients, and 16.51% were under the age of 60. The probability of adverse outcome was significantly higher in female participants (35.20%) than in male participants (24.33%) (Table 2).

**TABLE 2:** Participant characteristics by sex.

Variables	Sex		*P*
	Male	Female	
Participants	1,163	733	
RBC (10^12/L)	4.48 ± 0.62	4.11 ± 0.56	< 0.001
HGB (g/dL)	14.04 ± 1.93	12.68 ± 1.66	< 0.001
HCT (%)	41.56 ± 5.39	37.98 ± 4.69	< 0.001
WBC (10^9/L)	8.24 ± 2.83	8.00 ± 2.96	0.071
PLT (10^9/L)	219.29 ± 68.43	231.51 ± 74.33	< 0.001
TC (mg/dL)	175.18 ± 42.76	186.45 ± 44.54	< 0.001
TG (mg/dL)	96.00 (72.00–133.00)	92.00 (68.00–124.00)	0.005
HDL-c (mg/dL)	42.48 ± 15.89	47.04 ± 17.77	< 0.001
LDL-c (mg/dL)	101.93 ± 40.86	108.17 ± 44.42	0.002
BUN (mg/dL)	18.00 ± 8.57	16.63 ± 8.22	< 0.001
Scr (mg/dL)	0.97 (0.84–1.15)	0.74 (0.63–0.90)	< 0.001
FBG (mmol/L)	99.81 ± 46.28	97.87 ± 45.63	0.372
HBA1c (%)	5.80 (5.30–6.40)	5.90 (5.40–6.50)	0.206
PTINR	1.03 ± 0.28	1.06 ± 0.40	0.026
APTT (sec)	30.97 ± 5.96	30.56 ± 7.31	0.184
FIB (mg/L)	331.24 ± 92.27	328.54 ± 90.91	0.532
BMI (kg/m^2)	23.64 ± 3.05	23.30 ± 3.54	0.029
NIHSS score at admission	3.00 (1.00–6.00)	4.00 (1.00–9.00)	< 0.001
Age (years)			< 0.001
<60	313 (26.91%)	121 (16.51%)	
60 to < 70	325 (27.94%)	178 (24.28%)	
70 to < 80	393 (33.79%)	272 (37.11%)	
≥ 80	132 (11.35%)	162 (22.10%)	
Previous stroke/TIA	243 (20.89%)	158 (21.56%)	0.731
Hypertension	714 (61.39%)	492 (67.12%)	0.012
Diabetes	368 (31.64%)	240 (32.74%)	0.617
Hyperlipidemia	402 (34.57%)	295 (40.25%)	0.012
Smoking	708 (60.88%)	40 (5.46%)	< 0.001
Atrial fibrillation	229 (19.69%)	177 (24.15%)	0.021
CHD	144 (12.38%)	74 (10.10%)	0.129
mRS at admission			0.116
<3	1,036 (89.16%)	636 (86.77%)	
≥3	126 (10.84%)	97 (13.23%)	
Stroke etiology			0.004
LAA	380 (32.67%)	224 (30.56%)	
SVO	234 (20.12%)	130 (17.74%)	
CE	275 (23.65%)	217 (29.60%)	
Other determined	92 (7.91%)	75 (10.23%)	
Undetermined	182 (15.65%)	87 (11.87%)	
Probability of adverse outcome			< 0.001
No	880 (75.67%)	475 (64.80%)	
Yes	283 (24.33%)	258 (35.20%)	

Continuous variables: mean (standard deviation) or median (range). Categorized variables: N (%). HCT, hematocrit; AIS, Acute ischemic stroke; RBC, Red Blood Cell Count; HGB, Hemoglobin; WBC, White Blood Cell Count; PLT, Platelet Count; TC, Total Cholesterol; TG, Triglyceride; HDL-c, High-Density Lipoprotein-Cholesterol; LDL-c, Low-Density Lipoprotein-Cholesterol; BUN, Blood Urea Nitrogen; Scr, Serum Creatinine; FBG, Fasting Blood Glucose; HbA1c, Hemoglobin A1c; BMI, Body Mass Index; PTINR, International Normalized Ratio; APTT, Activated Partial Thromboplastin Time; FIB, Fibrinogen; TIA, Transient Ischemic Attack; NIHSS, National Institutes of Health Stroke Scale; CHD, Coronary Heart Disease; mRS, Modified Rankin Scale; LAA, large artery atherosclerosis; SVO, small vessel occlusion; CE, cardio embolism.

### 3.2 Multivariate analyses

We investigated the association between HCT levels and adverse outcome in AIS patients (Table 3). Overall, a 1% increase in HCT was associated with a 4% lower risk of adverse outcome (OR = 0.96, 95% CI 0.93–0.99, *P* = 0.0047). Compared to the lowest HCT quartile (Q1), the risk was 35% lower in Q2 (OR = 0.65, 95% CI 0.46–0.91, *P* = 0.0133) and 46% lower in Q3 (OR = 0.54, 95% CI 0.37–0.80, *P* = 0.0019), with a 22% lower risk in the highest quartile Q4 (OR = 0.78, 95% CI 0.52–1.17, *P* = 0.2297). In males, a 1% increase in HCT was associated with a 5% lower risk (OR = 0.95, 95% CI 0.91–0.98, *P* = 0.0019), with 52, 58, and 39% lower risks in Q2, Q3, and Q4 respectively. However, in females, HCT levels were not significantly associated with adverse outcome (OR = 0.99, 95% CI 0.94–1.04, *P* = 0.5989) (Table 3).

**TABLE 3:** Relationship between HCT and 3-month adverse outcome after AIS in different models.

Exposure	Crude model (OR, 95%CI, P)	Model I (OR, 95%CI, P)	Model II (OR, 95%CI, P)
**Male**			
HCT (%)	0.93 (0.91, 0.96) < 0.0001	0.94 (0.92, 0.96) < 0.0001	0.95 (0.91, 0.98) 0.0019
**HCT (%) quartile**			
Q1 (23.60–36.90)	1.0 (Reference)	1.0 (Reference)	1.0 (Reference)
Q2 (37.00–40.50)	0.59 (0.39, 0.89) 0.0123	0.56 (0.37, 0.86) 0.0081	0.48 (0.28, 0.81) 0.0065
Q3 (40.60–43.80)	0.40 (0.27, 0.60) < 0.0001	0.43 (0.28, 0.64) < 0.0001	0.42 (0.24, 0.71) 0.0013
Q4 (43.90–55.80)	0.46 (0.32, 0.66) < 0.0001	0.54 (0.37, 0.79) 0.0016	0.61 (0.37, 1.03) 0.0659
P for trend	0.0001	0.0022	0.1701
**Female**			
HCT (%)	0.95 (0.92, 0.98) 0.0012	0.96 (0.93, 1.00) 0.0292	0.99 (0.94, 1.04) 0.5989
**HCT (%) quartile**			
Q1 (23.60–36.90)	1.0 (Reference)	1.0 (Reference)	1.0 (Reference)
Q2 (37.00–40.50)	0.67 (0.47, 0.96) 0.0308	0.70 (0.48, 1.01) 0.0599	0.77 (0.48, 1.25) 0.2938
Q3 (40.60–43.80)	0.44 (0.28, 0.69) 0.0003	0.55 (0.35, 0.88) 0.0119	0.67 (0.36, 1.22) 0.1872
Q4 (43.90–55.80)	0.85 (0.49, 1.49) 0.5749	0.99 (0.56, 1.77) 0.9758	0.94 (0.43, 2.04) 0.8726
P for trend	0.0128	0.1422	0.4134
**Total**			
HCT (%)	0.94 (0.92, 0.96) < 0.0001	0.95 (0.93, 0.97) < 0.0001	0.96 (0.93, 0.99) 0.0047
**HCT (%) quartile**			
Q1 (23.60–36.90)	1.0 (Reference)	1.0 (Reference)	1.0 (Reference)
Q2 (37.00–40.50)	0.64 (0.49, 0.84) 0.0012	0.64 (0.48, 0.84) 0.0014	0.65 (0.46, 0.91) 0.0133
Q3 (40.60–43.80)	0.43 (0.32, 0.58) < 0.0001	0.49 (0.36, 0.66) < 0.0001	0.54 (0.37, 0.80) 0.0019
Q4 (43.90–55.80)	0.54 (0.40, 0.73) < 0.0001	0.66 (0.49, 0.90) 0.0089	0.78 (0.52, 1.17) 0.2297
P for trend	< 0.0001	0.0011	0.1364

Crude model adjust for: None. Model I adjust for: Age and Sex. Model II adjust for: WBC, PLT, TC, TG, HDL-c, LDL-c, BUN, Scr, FBG, HBA1c, PTINR, APTT, FIB, Age, Sex, BMI, Previous stroke/TIA, Hypertension, Diabetes, Hyperlipidemia, Smoking, Atrial fibrillation, CHD, NIHSS score at admission, mRS at admission, Stroke etiology. Sex was not adjusted in sex subgroups. HCT, hematocrit; AIS, Acute ischemic stroke; WBC, White Blood Cell Count; PLT, Platelet Count; TC, Total Cholesterol; TG, Triglyceride; HDL-c, High-Density Lipoprotein-Cholesterol; LDL-c, Low-Density Lipoprotein-Cholesterol; BUN, Blood Urea Nitrogen; Scr, Serum Creatinine; FBG, Fasting Blood Glucose; HbA1c, Hemoglobin A1c; BMI, Body Mass Index; PTINR, International Normalized Ratio; APTT, Activated Partial Thromboplastin Time; FIB, Fibrinogen; TIA, Transient Ischemic Attack; NIHSS, National Institutes of Health Stroke Scale; CHD, Coronary Heart Disease; mRS, Modified Rankin Scale.

### 3.3 The nonlinearity addressed by the generalized additive model

**FIGURE 3:**
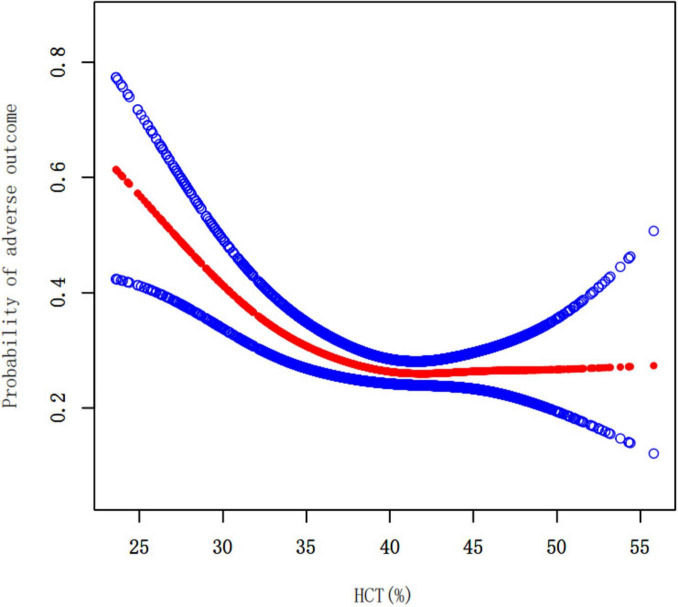
The nonlinear relationship between HCT and the probability of adverse outcome in overall population. A threshold, nonlinear association was found in a generalized additive model (GAM). Adjusting for WBC, PLT, TC, TG, HDL-c, LDL-c, BUN, Scr, FBG, HBA1c, PTINR, APTT, FIB, Age, Sex, BMI, Previous stroke/TIA, Hypertension, Diabetes, Hyperlipidemia, Smoking, Atrial fibrillation, CHD, NIHSS score at admission, mRS at admission, Stroke etiology.

**FIGURE 4:**
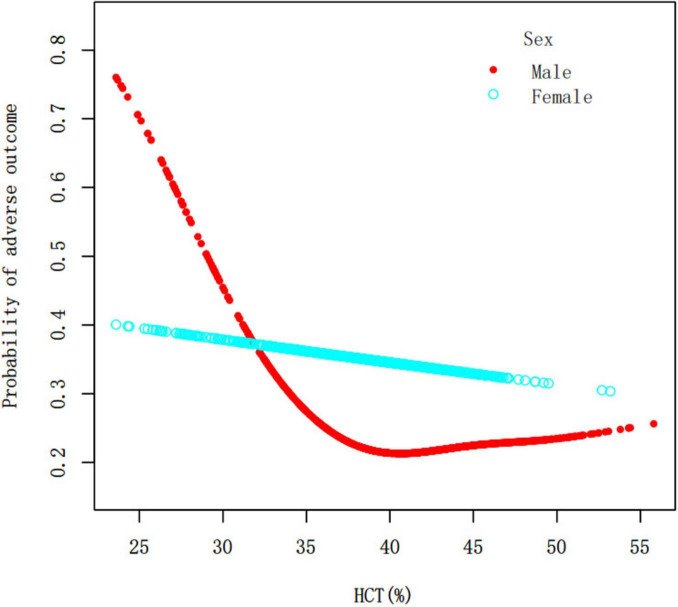
The nonlinear relationship between HCT and the probability of adverse outcome in males. Adjusting for WBC, PLT, TC, TG, HDL-c, LDL-c, BUN, Scr, FBG, HBA1c, PTINR, APTT, FIB, Age, BMI, Previous stroke/TIA, Hypertension, Diabetes, Hyperlipidemia, Smoking, Atrial fibrillation, CHD, NIHSS score at admission, mRS at admission, Stroke etiology.

There was a nonlinear association between HCT and the likelihood of a poor outcome in overall population (Figure 3 and Table 4). In overall population, for HCT levels below 34.4%, each 1% increase in HCT was associated with a 15% lower risk of adverse outcome (OR = 0.85, 95% CI 0.78–0.92, *P* = 0.0001) (Table 4). Further analysis using the two-piecewise linear regression model revealed sex-specific differences in the HCT-outcome relationship. In men, there was a nonlinear association between HCT and the chance of an unfavorable outcome (Figure 4 and Table 4). In males, for HCT levels below 36.4%, each 1% increase in HCT was associated with a 19% lower risk of adverse outcome (OR = 0.81, 95% CI 0.74–0.88, *P* < 0.0001). However, for HCT above 36.4%, there was no significant association (OR = 1.01, 95% CI 0.96–1.06, *P* = 0.6093) (Table 4). In females, HCT levels were not significantly associated with the risk of adverse outcome (Figure 4 and Table 4).

**TABLE 4:** The result of two-piecewise linear regression model.

Probability of adverse outcome	Male (OR, 95%CI, P)	Female (OR, 95%CI, P)	Total (OR, 95%CI, P)
Standard linear regression	0.95 (0.91, 0.98) 0.0019	0.99 (0.94, 1.04) 0.5989	0.96 (0.93, 0.99) 0.0047
**Two-piecewise linear regression**			
Inflection point of HCT (95% CI, %)	36.4 (32.2, 38.6)	41.3 (40.1, 42.8)	34.4 (29.8, 34.9)
≤ Inflection point	0.81 (0.74, 0.88) < 0.0001	0.97 (0.91, 1.03) 0.3146	0.85 (0.78, 0.92) 0.0001
> Inflection point	1.01 (0.96, 1.06) 0.6093	1.06 (0.91, 1.24) 0.4320	1.01 (0.96, 1.03) 0.8933
P for log-likelihood ratio test	< 0.001	0.329	0.002

Adjusting for WBC, PLT, TC, TG, HDL-c, LDL-c, BUN, Scr, FBG, HBA1c, PTINR, APTT, FIB, Age, Sex, BMI, Previous stroke/TIA, Hypertension, Diabetes, Hyperlipidemia, Smoking, Atrial fibrillation, CHD, NIHSS score at admission, mRS at admission, Stroke etiology. Sex was not adjusted in sex subgroups. HCT, hematocrit; AIS, Acute ischemic stroke; WBC, White Blood Cell Count; PLT, Platelet Count; TC, Total Cholesterol; TG, Triglyceride; HDL-c, High-Density Lipoprotein-Cholesterol; LDL-c, Low-Density Lipoprotein-Cholesterol; BUN, Blood Urea Nitrogen; Scr, Serum Creatinine; FBG, Fasting Blood Glucose; HbA1c, Hemoglobin A1c; BMI, Body Mass Index; PTINR, International Normalized Ratio; APTT, Activated Partial Thromboplastin Time; FIB, Fibrinogen; TIA, Transient Ischemic Attack; NIHSS, National Institutes of Health Stroke Scale; CHD, Coronary Heart Disease; mRS, Modified Rankin Scale.

### 3.4 Sensitivity analyses

We conducted sensitivity analyses to support our findings. Model I covered people without CHD, Model II included subjects without hypertension, and Model III included subjects without atrial fibrillation. In males with HCT levels below the inflection point, each 1% rise in HCT was linked with a decreased probability of poor outcome. However, for HCT over the inflection point, there was no significant relationship. All models corroborated the key findings of a nonlinear relationship in males, demonstrating the strength of our analysis. Female HCT levels were not significantly linked with the likelihood of poor outcome (Table 5).

**TABLE 5:** The result of the two-piecewise linear regression model in different sensitivity analyses.

Probability of adverse outcome	Male (OR, 95%CI, P)	Female (OR, 95%CI, P)	Total (OR, 95%CI, P)
**Model I**			
Standard linear regression	0.93 (0.90, 0.97) 0.0005	0.98 (0.93, 1.03) 0.5065	0.95 (0.93, 0.98) 0.0017
**Two-piecewise linear regression**			
Inflection point of HCT (%)	35.6	41.3	34.5
≤ Inflection point	0.78 (0.71, 0.87) < 0.0001	0.96 (0.90, 1.02) 0.2223	0.83 (0.76, 0.91) < 0.0001
> Inflection point	1.01 (0.95, 1.06) 0.9551	1.08 (0.92, 1.27) 0.3756	1.01 (0.96, 1.04) 0.9137
P for log-likelihood ratio test	< 0.001	0.260	< 0.001
**Model II**			
Standard linear regression	0.91 (0.85, 0.97) 0.0054	0.96 (0.87, 1.07) 0.4869	0.93 (0.88, 0.98) 0.0060
**Two-piecewise linear regression**			
Inflection point of HCT (%)	38.3	31.2	38.3
≤ Inflection point	0.74 (0.64, 0.85) < 0.0001	1.34 (0.61, 2.94) 0.4688	0.84 (0.77, 0.92) 0.0003
> Inflection point	1.05 (0.95, 1.17) 0.3283	0.94 (0.84, 1.06) 0.3218	1.02 (0.94, 1.11) 0.6860
P for log-likelihood ratio test	< 0.001	0.374	0.010
**Model III**			
Standard linear regression	0.94 (0.90, 0.98) 0.0063	0.99 (0.93, 1.05) 0.7059	0.96 (0.93, 0.99) 0.0152
**Two-piecewise linear regression**			
Inflection point of HCT (%)	36.3	41	38.1
≤ Inflection point	0.80 (0.72, 0.88) < 0.0001	0.96 (0.89, 1.04) 0.3446	0.89 (0.84, 0.95) 0.0002
> Inflection point	1.02 (0.96, 1.08) 0.5530	1.09 (0.90, 1.32) 0.3658	1.02 (0.97, 1.08) 0.4009
P for log-likelihood ratio test	< 0.001	0.294	0.004

Model I: covered people without CHD (*n* = 1,678). Model II: covered people without hypertension (*n* = 690). Model III: covered people without Atrial fibrillation (*n* = 1,490). Adjusting for WBC, PLT, TC, TG, HDL-c, LDL-c, BUN, Scr, FBG, HBA1c, PTINR, APTT, FIB, Age, Sex, BMI, Previous stroke/TIA, Hypertension, Diabetes, Hyperlipidemia, Smoking, Atrial fibrillation, CHD, NIHSS score at admission, mRS at admission, Stroke etiology. Stratified variables were not adjusted. HCT, hematocrit; AIS, Acute ischemic stroke; WBC, White Blood Cell Count; PLT, Platelet Count; TC, Total Cholesterol; TG, Triglyceride; HDL-c, High-Density Lipoprotein-Cholesterol; LDL-c, Low-Density Lipoprotein-Cholesterol; BUN, Blood Urea Nitrogen; Scr, Serum Creatinine; FBG, Fasting Blood Glucose; HbA1c, Hemoglobin A1c; BMI, Body Mass Index; PTINR, International Normalized Ratio; APTT, Activated Partial Thromboplastin Time; FIB, Fibrinogen; TIA, Transient Ischemic Attack; NIHSS, National Institutes of Health Stroke Scale; CHD, Coronary Heart Disease; mRS, Modified Rankin Scale.

We calculated an E-value to evaluate the sensitivity to unmeasured confounding. The major findings were robust, except for an unmeasured confounder with an OR larger than 1.46.

## 4 Discussion

This secondary analysis investigated the association between HCT and 3-month outcome in AIS patients. We identified a sex-specific nonlinear relationship, with male patients demonstrating an inflection point at 36.4% HCT—below this threshold, lower HCT levels were significantly associated with increased poor outcome risk. Conversely, female AIS patients showed no significant associations between HCT and 3-month outcome.

The association between HCT and outcome in patients with AIS remains a topic of considerable debate. Several studies have indicated that elevated HCT may play a significant role in determining recovery outcome. Sacco et al. discovered that elevated HCT might be an independent predictor of early mortality in women with AIS (11). Similarly, Tanné et al. demonstrated that elevated HCT levels were linked to poorer outcome (12). Furthermore, the relationship between HCT and stroke outcome appears to be complex, with some research revealing a U-shaped association. For example, Gotoh et al. found that both low and elevated HCT levels might increase the risk of stroke (13).

Conversely, low HCT levels have been associated with poor outcome. Research has demonstrated that low HCT levels ( < 27%) are linked to increased early post-stroke mortality, as evidenced by Sico et al. (14) and further supported by Hashem et al. (15). In a case-control study of 30 subjects, Hashem et al. observed that lower HCT levels were associated with poorer functional outcome (15). Kellert et al. found in a cohort analysis of 217 patients who received intravenous thrombolysis that low HCT levels were significantly associated with poor outcome at 3 months (mRS score ≥ 3) and mortality following AIS (5). These findings from Kellert et al. are consistent with our investigation. While Kellert et al. (5) also examined the relationship between HCT and AIS outcome, their study focused on patients undergoing endovascular treatment with a smaller sample size (217 cases) and employed only linear regression analysis, which may not have detected the nonlinear relationship between HCT and outcome observed in our study. Our investigation encompasses a broader population of AIS patients and adjusts for various potential confounding factors, including prior stroke history, stroke etiology, hypertension, and diabetes.

The identification of a threshold effect at HCT 36.4% in male AIS patients represents a critical pathophysiological transition point. This threshold defines an optimal oxygen transport window: below 36.4%, each 1% HCT increase significantly improves outcome (OR = 0.81, 95% CI 0.74–0.88) by enhancing cerebral oxygen delivery without excessive viscosity (39, 40). Above this inflection point, rising blood viscosity exponentially impairs microvascular perfusion in the ischemic penumbra where autoregulation is already compromised, negating the benefits of increased oxygen-carrying capacity (41, 42). Additionally, HCT levels below the threshold often indicate systemic inflammation and impaired erythropoiesis (10, 15), driving poor outcome through enhanced proinflammatory cytokines and tissue hypoxia (10, 43). Conversely, maintaining HCT above 36% mitigates these inflammatory cascades while avoiding the prothrombotic state associated with polycythemia (44, 45). This threshold thus represents a “Goldilocks zone” optimizing oxygen delivery without triggering hyperviscosity-related complications.

The absence of HCT-outcome associations in females despite higher adverse event rates (35.20% vs. 24.33%) reflects sex-specific biological mechanisms. Even in our predominantly postmenopausal cohort (83.49% ≥ 60 years), residual estrogenic activity fundamentally alters cerebrovascular physiology through eNOS upregulation and enhanced vasodilation, rendering cerebral perfusion less dependent on hematocrit-mediated oxygen transport optimization and effectively buffering the threshold effect observed in males (20, 21). Additionally, women demonstrate distinct inflammatory profiles with higher anti-inflammatory cytokines (IL-10) and different complement activation patterns that create alternative pathophysiological pathways overshadowing HCT effects (22), while intrinsic differences in red blood cell deformability and plasma viscosity characteristics establish sex-specific optimal HCT ranges that differ substantially from males (23).

The clinical significance of this secondary analysis lies in its identification of potential sex-specific nonlinear associations between HCT levels and 3-month outcome in AIS patients, providing new insights into stroke research and highlighting the importance of sex-stratified approaches in outcome assessment. Our findings suggest that in male patients, HCT levels below 36.4% are significantly associated with an increased risk of poor outcome. Furthermore, future research could explore the mechanisms by which HCT functions in patients of different sexes and how it can be integrated into personalized treatment plans to improve stroke outcome.

### 4.1 Study strengths and limitations

This study has several strengths. First, we conducted a secondary analysis of a well-designed prospective cohort study with comprehensive clinical data on AIS patients, including demographic characteristics, medical history, and laboratory indicators, which provides more representative and reliable evidence. Second, we utilized advanced statistical analysis methods, such as GAM and piecewise linear regression, which can more accurately capture the nonlinear relationship between HCT and outcome rather than being limited to simple linear analysis. Furthermore, this study conducted in-depth analyses of sex differences, revealing that the relationship between HCT and outcome differs significantly between male and female patients. This finding not only enriches the existing literature but also provides new insights for clinical practice, suggesting the potential need for individualized HCT assessment strategies based on sex. We also verified the robustness of our research findings through sensitivity analyses, further enhancing the reliability of the conclusions.

This work has several limitations. First, only South Korean patients were included in this study. Therefore, further validation is needed for our findings across different geographic and ethnic populations. Second, certain variable information lacked sufficient detail. For instance, rather than providing exact ages, the original study database contained age-stratified data in 10-year increments, which may have resulted in inadequate granularity for these factors. Future studies should consider more precise data collection methods to obtain detailed variable information. Third, although recognized potential confounders were adjusted for, this study may contain unmeasured or uncontrolled confounding factors, as is common with all observational research. Multiple potential confounders such as lesion size, timing of HCT determination, time to treatment, and specific treatment received were not available in the original dataset and remain unaddressed in our analysis. To assess the potential impact of unmeasured confounders, we calculated the E-value and found it unlikely that unmeasured confounding could fully explain our findings.

## 5 Conclusion

In this secondary analysis of 1,896 AIS patients from an existing South Korean prospective cohort, we identified a nonlinear association between HCT and 3-month outcome with sex-specific patterns. Male patients demonstrated a significantly increased risk of poor outcome when HCT fell below the threshold, while no significant association was observed in females. Prospective validation through multicenter studies across diverse populations is needed to establish clinical utility.

## Data Availability

The original contributions presented in this study are included in this article/Supplementary material, further inquiries can be directed to the corresponding authors.

## Footnotes

1
https://doi.org/10.1371/journal.pone.0228738
