# Supplementary material for: Nonlinear association between hematocrit and 3-month outcome after acute ischemic stroke: identification of threshold effects in males
Source: Front Med (Lausanne). 2026 Feb 4;13:1721251. doi: 10.3389/fmed.2026.1721251 (PMC12913135; doi:10.3389/fmed.2026.1721251)
Supplement: Supplementary file 1 [file Table_1.docx]

Supplementary Material

# Supplementary Tables

Table S1 Collinearity screening

|  | Variance inflation factor | | |
| --- | --- | --- | --- |
|  | Step 1 | Step 2 | Step 3 |
| HCT (%) | 26.5 | 7.9 | 1.6 |
| RBC (10^12/L) | 7.9 | 7.9 | NA |
| HGB(g/dL) | 21.6 | NA | NA |
| WBC (10^9/L) | 1.2 | 1.2 | 1.2 |
| PLT (10^9/L) | 1.3 | 1.2 | 1.2 |
| TC (mg/dL) | 3 | 3 | 3 |
| TG (mg/dL) | 1.3 | 1.2 | 1.2 |
| HDL-c(mg/dL) | 1.2 | 1.2 | 1.2 |
| LDL-c(mg/dL) | 3 | 3 | 3 |
| BUN (mg/dL) | 2.1 | 2.1 | 2.1 |
| Scr (mg/dL) | 2 | 2 | 2 |
| FBG (mmol/L) | 1.4 | 1.4 | 1.4 |
| HBA1c (%) | 1.4 | 1.4 | 1.4 |
| PTINR | 1.3 | 1.3 | 1.3 |
| APTT (sec） | 1.2 | 1.2 | 1.2 |
| FIB (mg/L) | 1.2 | 1.2 | 1.2 |
| Sex | 1.8 | 1.8 | 1.8 |
| Age(years) | 1.4 | 1.4 | 1.3 |
| BMI (kg/m^2) | 1.3 | 1.3 | 1.2 |
| Previous stroke/TIA | 1.1 | 1.1 | 1.1 |
| Hypertension | 1.2 | 1.2 | 1.2 |
| Diabetes | 1.5 | 1.5 | 1.4 |
| Hyperlipidemia | 1.2 | 1.2 | 1.2 |
| Smoking | 1.5 | 1.5 | 1.5 |
| Atrial fibrillation | 1.3 | 1.3 | 1.3 |
| CHD | 1.1 | 1.1 | 1.1 |
| NIHSS score at admission | 1.3 | 1.3 | 1.3 |
| mRS at admission | 1.1 | 1.1 | 1.1 |
| Stroke etiology | 1.1 | 1.1 | 1.1 |

Variance inflation factor = 1/(1-R^2^).

The variables with Variance inflation factor >5 will be regarded as collinear variables.

**NA was the excluded variable**

HGB and RBC were excluded

| HCT: hematocrit, AIS: Acute ischemic stroke, WBC: White Blood Cell Count, PLT: Platelet Count, TC: Total Cholesterol, TG: Triglyceride, HDL-c: High-Density Lipoprotein-Cholesterol, LDL-c: Low-Density Lipoprotein-Cholesterol, BUN: Blood Urea Nitrogen, Scr: Serum Creatinine, FBG: Fasting Blood Glucose, HbA1c: Hemoglobin A1c, BMI: Body Mass Index, PTINR: International Normalized Ratio, APTT: Activated Partial Thromboplastin Time, FIB: Fibrinogen, TIA: Transient Ischemic Attack, NIHSS: National Institutes of Health Stroke Scale, CHD: Coronary Heart Disease, mRS: Modified Rankin Scale. |
| --- |
